# Understanding the public’s role in reducing low-value care: a scoping review

**DOI:** 10.1186/s13012-020-00986-0

**Published:** 2020-04-07

**Authors:** Emma E. Sypes, Chloe de Grood, Fiona M. Clement, Jeanna Parsons Leigh, Liam Whalen-Browne, Henry T. Stelfox, Daniel J. Niven

**Affiliations:** 1 Department of Community Health Sciences, Cumming School of Medicine, University of Calgary, Calgary, Canada; 2 O’Brien Institute of Public Health, Cumming School of Medicine, University of Calgary, Calgary, Canada; 3 School of Health Administration, Faculty of Health, Dalhousie University, Calgary, Canada; 4 Department of Critical Care Medicine, Cumming School of Medicine, University of Calgary and Alberta Health Services, Calgary, Canada

**Keywords:** Low-value care, De-implementation, De-adoption, Patient engagement, Choosing wisely

## Abstract

**Background:**

Low-value care initiatives are rapidly growing; however, it is not clear how members of the public should be involved. The objective of this scoping review was to systematically examine the literature describing public involvement in initatives to reduce low-value care.

**Methods:**

Evidence sources included MEDLINE, EMBASE, and CINAHL databases from inception to November 26, 2019, grey literature (CADTH Tool), reference lists of included articles, and expert consultation. Citations were screened in duplicate and included if they referred to the public’s perception and/or involvement in reducing low-value care. Public included patients or citizens without any advanced healthcare knowledge. Low-value care included medical tests or treatments that lack efficacy, have risks that exceed benefit, or are not cost-effective. Extracted data pertained to study characteristics, low-value practice, clinical setting, and level of public involvement (i.e., patient-clinician interaction, research, or policy-making).

**Results:**

The 218 included citations were predominantly original research (*n* = 138, 63%), published since 2010 (*n* = 192, 88%), originating from North America (*n* = 146, 67%). Most citations focused on patient engagement within the patient-clinician interaction (*n* = 156, 72%), using tools that included shared decision-making (*n* = 66, 42%) and patient-targeted educational materials (*n* = 72, 46%), and reported both reductions in low-value care and improved patient perceptions regarding low-value care. Fewer citations examined public involvement in low-value care policy-making (*n* = 33, 15%). Among citations that examined perspectives regarding public involvement in initiatives to reduce low-value care (*n* = 10, 5%), there was consistent support for the utility of tools applied within the patient-clinician interaction and less consistent support for involvement in policy-making.

**Conclusions:**

Efforts examining public involvement in low-value care concentrate within the patient-clinician interaction, wherein patient-oriented educational materials and shared decision-making tools have been commonly studied and are associated with reductions in low-value care. This contrasts with inclusion of the public in low-value care policy decisions wherein tools to promote engagement are less well-developed and involvement not consistently viewed as valuable.

**Trial registration:**

Open Science Framework (https://osf.io/6fsxm)

Contributions to the literature

- Low-value care initiatives are rapidly growing; however, it is not clear how members of the public should be involved.
- Our study identified that efforts examining public involvement in low-value care concentrate within the patient-clinician interaction, wherein patient-oriented educational materials and shared decision-making tools have been commonly studied and are associated with reductions in low-value care.
- Tools to promote inclusion of the public in low-value care policy decisions are less well-developed, and involvement is not consistently viewed as valuable.

## Background

The ongoing use of low-value healthcare practices (i.e., low-value care), broadly defined as medical tests or treatments that lack efficacy, have risks that exceed benefit, or are not cost effective [1], impedes the delivery of safe, efficient, and cost-effective healthcare [2]. For patients and their caregivers, receiving a low-value test or treatment can lead to physical, psychological, and financial consequences [2–4]. Estimates suggest that unnecessary care in the USA costs upwards of $210 billion dollars annually [5] and consumes resources that could be allocated to high-value, necessary care. Studies from Australia and the USA identified 156 [6] and 146 [7] low-value practices, respectively, and over 500 “Do Not Do” recommendations have been produced through the Choosing Wisely campaign [8]. In the UK, the National Institute for Health and Care Excellence (NICE) has included upwards of 1000 “Do Not Do” recommendations in their evidence-based guidelines for care in the National Health Service (NHS) [9]. However, this identification of low-value practices has not been followed by a commensurate reduction in their use [10–12]. This is likely influenced by a number of factors [13], one of which may be challenges with engaging relevant stakeholders such as clinicians, decision makers, and the public.

Members of the public (e.g., patients, caregivers, and citizens) have been identified as important stakeholders within initiatives to reduce low-value care [1, 14, 15]. Their dual role in this process includes (1) payment for healthcare services directly or indirectly and (2) recipients of healthcare as patients. Thus, there are multiple opportunities for their inclusion in efforts to reduce low-value care. At its inception, the Choosing Wisely campaign recognized the patient-clinician interaction as an opportunity to reduce low-value care and developed patient-targeted educational materials to empower patients to engage with their clinicians in a joint effort to avoid selected low-value care practices [16]. Patient and public representatives may also contribute to research activities aiming to reduce low-value care, as numerous patient-targeted interventions continue to be developed and evaluated [17]. In addition, there has been a call for public involvement within healthcare policy and administration, with opportunities in health technology re-assessment [18] and health system-level initiatives aiming to reduce low-value care [1, 19].

How patients and the public are optimally involved in initiatives to reduce low-value care has been highlighted as a deficiency in the science that underpins reducing low-value care [15, 20]. While a number of reviews and editorials speak to engaging the public in reducing low-value care [1, 21–23], there is a poor understanding of which organizations and stakeholders should engage the public, the extent to which the public should be involved, and how public involvement impacts initiatives to reduce low-value care, and importantly, how members of the public themselves wish to be involved. Given these broad knowledge gaps, we used scoping review methodology to systematically examine the literature to further understand current strategies for public involvement in reducing low-value care and identify areas that require additional research. Scoping review methodology was selected as it provides the optimal approach to synthesizing and mapping evidence from a body of literature that is predicted to be large and heterogenous [24, 25].

## Methods

### Overview and definitions

Methods were guided by the Joanna Brigg’s Institute Methodology for Scoping Reviews [24], and the protocol was registered with the Open Science Framework (https://osf.io/6fsxm). The Preferred Reporting Items for Systematic Reviews and Meta-Analyses extension for Scoping Reviews (PRISMA-ScR) checklist was used to guide reporting of methods and findings [25]. Operational definitions for the terms “low-value care,” “public,” and “public involvement” are presented in Table 1. Our operational definition for “low-value care” was based on that proposed by Elshaug et al. as a medical test or treatment “in which evidence suggests it confers no or very little benefit for patients, or risk of harm exceeds probable benefit, or, more broadly, the added costs of the intervention do not provide proportional added benefit” [1]. Although other definitions of low-value care exist, owing to a lack of consensus within the literature [17], we chose this definition because it is broad and encompasses three concepts commonly used when conceptualizing the value of care (i.e., cost, efficacy, and safety).

**Table 1:** Operational definitions for key concepts

Term	Operational definition	Example
Low-value care	Medical tests and treatments that meet one or more of the following criteria: lack of efficacy, not cost-effective, or risks exceeded benefit	Antibiotics for viral upper respiratory tract infections
Public	Patients, caregivers, and potential patients without advanced healthcare knowledge	A patient attending an appointment at a primary care clinic
	Excludes clinicians (any front-line healthcare professional), healthcare researchers, and healthcare administrators	
Public involvement	The engagement of members of the public in an initiative aiming to reduce low-value care	Engaging a patient in shared decision-making to explore their preferences and the potential risks and benefits to a low-value diagnostic imaging test

### Data sources and searches

We searched MEDLINE, EMBASE, and CINAHL databases from inception to November 26, 2019. The initial search was conducted on June 28, 2018, then updated on November 26, 2019. The search strategy (Additional file 1) was developed in consultation with a medical librarian and was peer reviewed by a second medical librarian using the Peer Review of Electronic Search Strategies (PRESS) checklist [26]. Search terms included keywords and their synonyms relevant to three main concepts: low-value care, the public, and public involvement. Recognizing that terminology may be nuanced, low-value care literature was identified using the most commonly cited terminology within contemporary scientific literature (e.g., de-adoption, overuse, and de-implementation) [27, 28]. The search terms were inclusive of all common terms identified in a prior scoping review of the literature [27]. These intentionally broad search terms acknowledge the absence of a universally agreed-upon taxonomy of terms that refer to low-value care and established medical subject heading terms to identify low-value care articles. The database search was limited to English as much of the terminology pertaining to low-value care (e.g., Choosing Wisely, low value, and overuse) is language-specific and may not translate well across languages. Given the broad nature of the research question, there was no limitation of the search strategy based on the study design. Additional citations were identified by searching the grey literature using the Canadian Agency for Drugs and Technologies in Health (CADTH) tool [29] (Additional file 2), reference lists of included articles, and consultation with experts in the field.

### Citation selection and screening

Citations were eligible for inclusion if they were written in English and referred to the public’s perception of and/or direct involvement in reducing low-value care. All study designs were eligible for inclusion. Citations were excluded if they predominantly focused on clinician-targeted strategies for reducing low-value care (e.g., personalized audit and feedback data). Eligible citations were screened independently in two steps by two investigators using Endnote (Clarivate Analytics, Philadelphia, USA). Prior to screening, the citation screening form was pilot tested using a random sample of 50 citations. The form was refined until agreement was consistent as denoted by a kappa statistic (*k*) > 0.8. During level one screening, both investigators examined the title and abstract of each citation to determine its eligibility for full text review. Citations that met the eligibility criteria or were unclear proceeded to level two screening, where both investigators reviewed the full text of each citation to determine eligibility. If the citation was excluded, the precise reason for exclusion was recorded. For citations without abstracts, the title was used to assess for eligibility at title/abstract screening, and if the title appeared relevant, the citation proceeded to full-text screening. Reference lists of included articles were screened in a similar fashion, first, by title and then by full text both independently and in duplicate. Any disagreements were resolved through discussion or consultation with another author (DJN). Agreement during both phases of screening was quantified using the kappa statistic [30].

### Data extraction

All data extraction was conducted independently by two investigators using DistillerSR (Evidence Partners, Ottawa, Canada). We used a conceptual framework to guide data extraction (Table 2). Prior to full data extraction, our data extraction form was pilot tested using six randomly selected citations. Extracted data broadly pertained to study characteristics (e.g., year, country, and study design), the low-value practice of interest (i.e., diagnostic test or therapeutic treatment), and the clinical setting (e.g., emergency department and primary care). We mapped included citations to our conceptual framework (Table 2) to capture the phase of de-implementation in which the public was involved (e.g., identifying and prioritizing low-value practices for de-implementation) and extracted additional data to provide further detail about *how* and *where* the public was involved in reducing low-value care. To understand *how* the public was involved, we extracted data that described how the public was engaged in an initiative to reduce low-value care (e.g., shared decision-making). This is referred to as the “strategy for public involvement.” To understand *where* the public was involved, we categorized each citation by the “level of patient engagement,” which included the “patient-clinician interaction” (i.e., strategies for public involvement that were employed during a clinical interaction), “research” (i.e., involving the public in conducting or evaluating research aiming to reduce low-value care), or “policy/administration” (i.e., involving the public in policy or administration level initiatives to reduce low-value care). Because this was a scoping review wherein a large number of heterogeneous citations was expected and desired, quality assessment of included citations was felt to be unlikely to yield the kind of useful information that it would for a more focused systematic review; thus, in accord with the PRISMA extension for scoping reviews, quality assessment of included citations was not performed [25].

**Table 2:** Conceptual framework for data extraction

Phase of de-implementation^a^	Operational definition	Example
Identify and prioritize low-value clinical practices	1) The public’s conceptual understanding of low-value care 2) The public’s involvement in identifying or prioritizing low-value practices for de-implementation	1) A survey asking members of the public to describe low-value care 2) Patient and provider co-creation of a priority list of practices for de-implementation
Assess barriers and facilitators to de-implementation	The public’s perception of barriers and facilitators to reducing low-value care	Exploring patient perspectives on the demand for low-value care
Select, tailor, and implement de-implementation intervention	Public involvement in developing interventions to reduce low-value care	Involving a patient representative in the design of an intervention to reduce a low-value practice
Evaluate de-implementation process and outcomes	The public’s involvement in the evaluation of outcomes of an initiative to reduce low-value care	Inclusion of patient-reported outcomes in an intervention to reduce the use of a low-value practice

^a^Adapted from Niven et al. model [27]

### Data synthesis and analysis

Included citations were mapped to a conceptual framework to describe how the public was engaged in reducing low-value care (Table 2). The framework was developed by determining which components of a conceptual framework for facilitating de-implementation were most relevant to public involvement [27]. Included citations that described or evaluated a strategy for public involvement were assessed to determine whether they indicated support or did not support the given strategy. For original research citations (e.g., randomized clinical trial), a statistically significant reduction in the targeted low-value aspect of care indicated support for the given public involvement strategy. For non-original research citations, (e.g., editorial) support was indicated by a generally positive discussion of the given strategy within the citation. All data was summarized by numerical counts and percentages as appropriate using the Stata statistical software (StataCorp, TX, USA).

## Results

### Citation selection

**Fig. 1:**
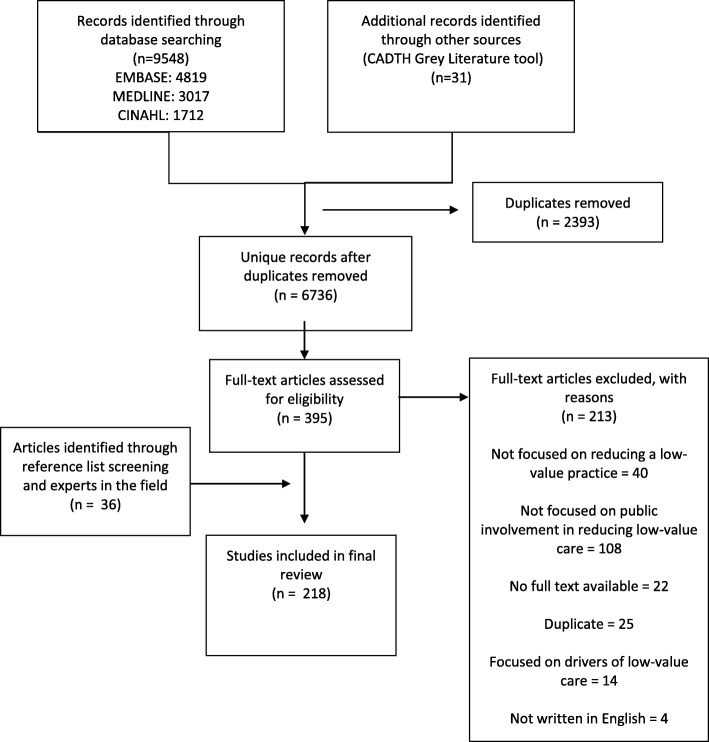
Selection of studies included in the review

Searches yielded 9548 citations from electronic databases and 31 citations from the grey literature (Fig. 1). After removing duplicates, 6736 unique citations were screened for inclusion from which 395 proceeded to full-text screening, and 182 were included in the review. The most common reasons for excluding citations during full-text screening were lack of focus on reducing a low-value practice and focus on other stakeholders such as physicians. Screening reference lists of included citations and consultation with experts identified an additional 36 citations which were included in the final review. Combined with the 182 citations, the final review included 218 citations. Most included citations derived from electronic databases (*n* = 160), followed by reference list/expert consultation (*n* = 36) and grey literature (n = 22).

### Study characteristics and classification within conceptual frameworks

**Fig. 2:**
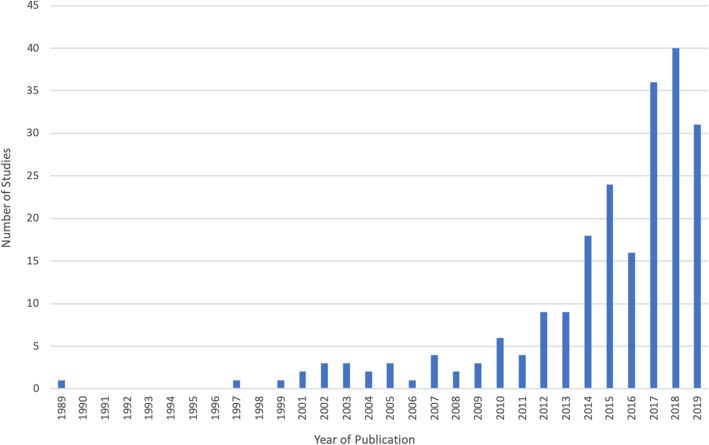
Year of publication of included studies (*n* = 218)

A detailed bibliography of included citations is available in Additional file 3, and an overall summary of relevant characteristics is presented in Table 3. Included citations were predominantly original research (*n* = 138, 63%) from North America (*n* = 146, 67%). Most citations were published in the last 10 years (*n* = 192, 88%), with a large increase following 2012/2013 (Fig. 2). Among citations reporting original research, most were observational studies (*n* = 34, 16%), qualitative designs (*n* = 28, 13%), or randomized clinical trials (*n* = 21, 10%). Other article types included narrative reviews (*n* = 34, 16%), commentaries (*n* = 34, 16%), and website items (e.g., medical society websites and health technology assessment websites) (*n* = 10, 5%). Most citations spoke of low-value care in a general sense (*n* = 95, 43%), with 32% (*n* = 69) focusing on low-value treatments and 17% (*n* = 38) on low-value tests. Among citations that reported reducing low-value care within a specific clinical setting, the most common location was within inpatient hospital departments (*n* = 42, 19%), followed by primary care practices (*n* = 35, 16%) and the community (*n* = 28, 13%).

**Table 3:** Characteristics of included citations (*n* = 218)

Characteristic	*N* (%)
Year of publication	
1980–1999	3 (1.4)
2000–2009	23 (10.5)
2010–2019	192 (88.1)
Continent of origin	
North America	146 (66.7)
Europe	41 (18.7)
Australia	20 (9.2)
Asia	8 (3.7)
Africa	1 (0.5)
South America	1 (0.5)
Oceania	1 (0.5)
Type of article	
Original Research	138 (63.3)
Observational^a^	34 (15.5)
Qualitative	28 (12.8)
Randomized controlled trials	21 (9.6)
Non-randomized experimental	13 (5.9)
Knowledge synthesis	12 (5.5)
Consensus method	11 (5.0)
Mixed methods	8 (3.7)
Community jury	8 (3.7)
Other^b^	3 (1.7)
Non-original research	80 (36.5)
Narrative review	34 (15.5)
Editorial/commentary	34 (15.5)
Website items	10 (4.5)
Policy report	2 (0.9)
Type of low-value care	
Low-value care in general	95 (43.4)
Specific low-value practice(s)	124 (56.6)
Test	38 (17.4)
Treatment	69 (31.5)
Both	16 (7.3)
Clinical setting	
Hospital	42 (19.2)
Primary care	35 (16.0)
Emergency Department	22 (10.0)
Community^c^	27 (12.4)
Not specified	92 (42.0)
Level of public engagement^d^	
Patient interaction	156 (71.6)
Research	56 (25.7)
Policy/administration	33 (15.1)

^a^Includes cohort, cross-sectional, and case-control studies

^b^Includes one case report and two public health outreach studies

^c^Includes outpatient clinics, long-term care homes, dentistry, and community pharmacies

^d^Describes where public involvement occurred. Clinical interaction: strategies for public involvement that were employed during a clinical interaction such as a primary care visit; research: involving the public in conducting or evaluating research aiming to reduce low-value care such as patient-reported outcomes; policy/administration: involving the public in policy or administration level initiatives to reduce low-value care, such as prioritizing practices for disinvestment

### Inclusion of the public in strategies that aim to reduce low-value care

**Fig. 3:**
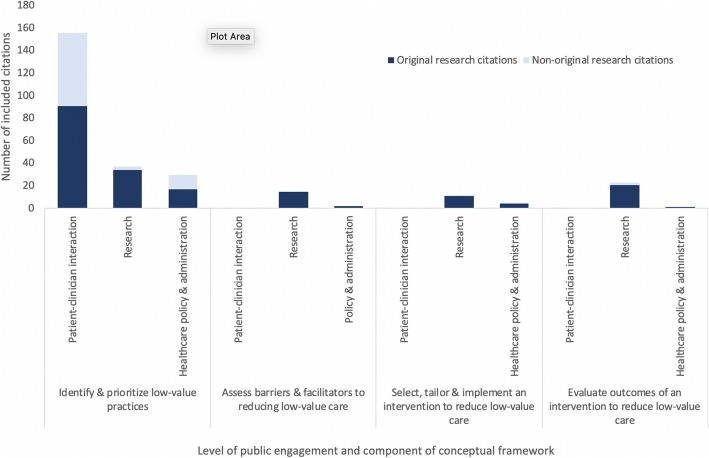
Classification of included studies (*n* = 218) according to level of public engagement and main components of the conceptual framework

**Fig. 4:**
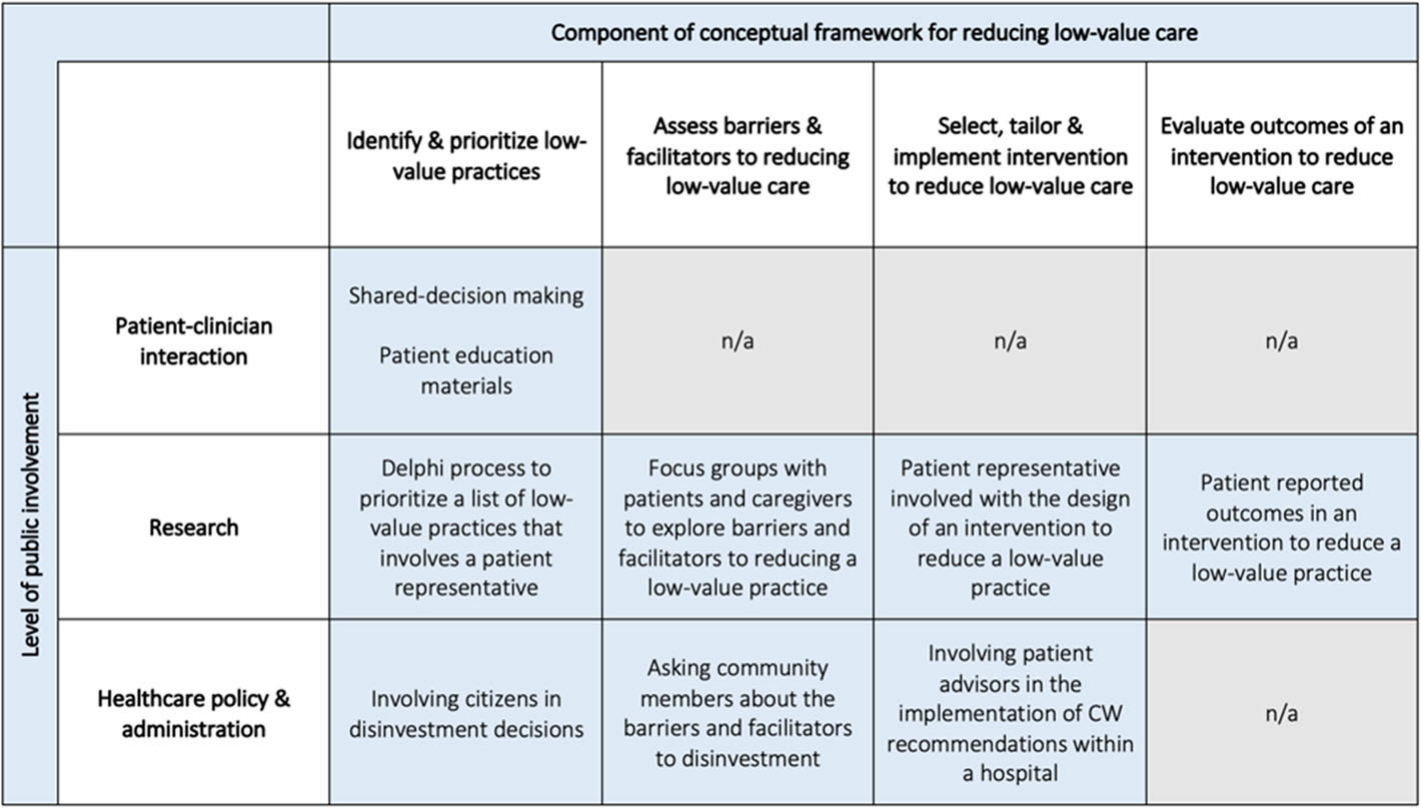
Strategies for public involvement in reducing low-value care identified from included citations, according to level of engagement and main components of the conceptual framework

**Fig. 5:**
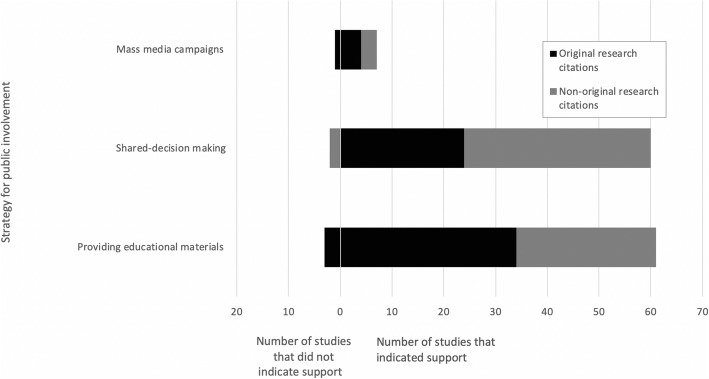
Reported utility of strategies for influencing decision-making about low-value care within the patient-clinician interaction

Strategies for public involvement in reducing low-value care were described or tested in 209 citations. Of these, 128 (61%) were original research, and 80 (38%) were non-original research. Most citations were referred to a strategy that engaged patients within the setting of a patient-clinician interaction (*n* = 148, 71%). A smaller number addressed inclusion of the public in policy/administrative decision-making (*n* = 31, 15%) or low-value care research (*n* = 56, 27%) (Fig. 3). Examples of common strategies for public involvement across all three levels of engagement and within their respective components of the conceptual framework for reducing low-value care are displayed in Fig. 4. At the patient-clinician interaction level, the focus was on helping patients identify low-value practices through the dissemination of educational materials and approaches such as shared decision-making (individual study details in Additional file 3). As depicted in Fig. 4, the significance of outcomes and/or discussion within these citations mostly indicated support for the utility of these strategies in reducing low-value care (“support” fully defined in the “Data Synthesis and Analysis” section). Of the 66 studies that focused on shared decision-making, 60 (91%) supported that tool as a means of engaging patients in reducing low-value care, of which 24 (40%) were original research. In studies that tested a shared decision-making approach, many reported improved patient knowledge and satisfaction with their decision-making process. In four studies (6%), it was unclear whether support for the given patient engagement strategy was positive or negative. Two studies (3%) did not support shared decision-making as a strategy for public engagement; however, these studies were both non-original research (website and narrative review). Of the 73 studies that discussed or evaluated providing educational materials as a strategy for public involvement, six (8%) did not comment on support for the strategy and three did not support the strategy (4%). Of the 64 remaining articles that did indicate support, 36 (56%) were original research, and 28 (44%) were non-original research (Fig. 5).

With regard to public involvement in policy-making relevant to low-value care, 28 of 31 (90%) citations referenced public involvement in identifying and prioritizing low-value practices, such as through the involvement of citizens in disinvestment decisions. Of these studies, most were original research (*n* = 20, 71%) and involved the public through surveys, focus groups, and community engagement events to solicit their perspective about de-implementation decisions. Fewer studies involved the public in assessing barriers and facilitators to reducing a low-value practice (*n* = 2, 6%) or in helping to develop an intervention to reduce a low-value practice (*n* = 5, 15%) (Fig. 3).

Within research activities, the public was engaged within all steps in our conceptual framework, from identifying and prioritizing low-value practices (*n* = 37, 66%) (e.g., involving patients in developing a Choosing Wisely list) to evaluating outcomes in interventions that aimed to reduce their use (*n* = 23, 41%) (e.g., evaluating patient satisfaction with care and decision-making process) (Fig. 3). Here, studies commonly engaged the public in the development of educational materials or other patient-targeted tools used within interventions so that they would be clear and effective for patient use.

### Stakeholder perceptions regarding public engagement in reducing low-value care

Ten citations examined stakeholder perspectives regarding public involvement in reducing low-value care (Table 4). Of these, five engaged demand-side stakeholders, including patients and other members of the public. The most commonly discussed strategy for public involvement was shared decision-making with care providers (*n* = 4 citations), wherein demand-side stakeholders unanimously agreed on its utility. One study from the UK asked community members if they thought citizens should be involved in disinvestment decision-making (i.e., the decision to withdraw resources from a given medical practice [41]) within the NHS, and the responses were overall negative [33]. In this study, community members felt as though citizens may not have the knowledge and expertise required to participate in disinvestment decision-making [33]. Another study from the UK posed the same question to healthcare administrators and found that while they were supportive of involving citizens, there were concerns about how to achieve meaningful engagement and the potential challenges that may arise [36]. Among the studies that engaged supply-side stakeholders, including physicians, nurses, and administrators, suggestions for public involvement that were supported included providing education about low-value practices [37, 38] and shared decision-making between care providers [37].

**Table 4:** Stakeholder perspectives on how the public should be involved in reducing low-value care

Study	Country	Study design	Stakeholder	Setting	Low-value practice(s)	Strategy for public involvement	Supportive of strategy?
**Demand-side stakeholders**^**a**^							
Kullgren [31] 2018	USA	Focus groups	Patients (seniors)	Not specified	Multiple (potentially harmful medications, cancer screening)	Shared decision-making	Yes; patients felt that understanding risks and benefits and reaching a personalized decision would help them support CW recommendations
Linsky [32] 2014	USA	Interviews and focus groups	Patients	Primary care	Unnecessary medications	Shared decision-making	Yes; main theme from focus groups and interviews was the importance of strong patient-provider relationships, trust, and SDM for reducing unnecessary medications
Hislop [33] 2011	UK	Interviews	Community members	Government	Low-value care in general	Citizen involvement in disinvestment decision-making	No; community members interviewed felt that taxpayers do not have the knowledge and impartiality required to be involved in decision-making
Rohrbacher [34] 2008	Germany	Telephone survey	Patients	Not specified	Low-value care in general	Shared decision-making	Yes; patients want to discuss their preferences and personal experiences with their physician to arrive at the most evidence-based decision
Schoenborn [35] 2017	USA	Interviews	Patients (seniors)	Ambulatory clinic	Cancer screening when life expectancy is limited	Shared decision-making	Yes; patients indicated preference for a trusting relationship with their physician and an individualized decision-making process
**Supply-side stakeholders**^**b**^							
Daniels [36] 2018	UK	Q study and interviews	Physicians and administrators	Government	Low-value care in general	Citizen Involvement in disinvestment decision-making	Yes (cautious); participants held an overall supportive but cautious stance to citizen involvement
Kanzaria [37] 2015	USA	Survey	ED physicians	ED	Advanced diagnostic imaging	Educating patients and families on low-probability outcomes and shared decision-making	Ninety-two percent of emergency physicians surveyed and indicated that SDM would be helpful in reducing low-value diagnostic imaging
Scales [38] 2017	USA	Survey	Physicians and nurses	Long-term care homes	Unnecessary antibiotics	Educating residents and families about why antibiotics are not necessary	Yes; survey findings supported education as a tool to help reduce unnecessary antibiotic use
Ellen [39] 2018	Israel	Interviews	Nurses	Outpatient clinic	Low-value care in general	Educating patients on the dangers of overuse	Yes; interview findings indicate that nurses support the need to educate patients about overuse
Embrett [40] 2018	Canada	Focus groups	Physicians	Not specified	Low-value care in general	Engaging patients in a conversation about low-value care in the clinical encounter	Yes; a principal finding from the focus groups was the need for the Choosing Wisely campaign to help facilitate patient conversations about low-value care during the clinical encounter

*ED* emergency department

^a^Stakeholders that receive health care (e.g., patients and community members)

^b^Stakeholders that contribute to the provision of health care (e.g., physicians and policy makers)

## Discussion

We identified a large number of citations that described, evaluated, or suggested a strategy for public involvement in reducing low-value care. The majority of included citations were published following inception of Choosing Wisely in 2012 [42]. Current literature suggests that public involvement in reducing low-value care takes place across three levels: (1) patient-clinician interactions, (2) policy/administrative decision-making, and (3) research. Most citations focused on the patient-clinician interaction. Patient-targeted educational tools and shared decision-making were commonly described or tested strategies that demonstrated utility in reducing low-value care and were supported by effected stakeholders. In policy-making and healthcare administration regarding low-value care, the most commonly cited role for the public was providing input on the prioritization of practices for de-implementation. However, the perceived utility of public involvement in these circumstances was questioned by both the public and healthcare administrators. Within low-value care research, examples of public involvement included developing patient-targeted tools to be used in de-implementation interventions and being a public representative in projects to reduce low-value care. Given the breadth of the literature examined, the importance of public inclusion as stakeholders in de-implementation science initiatives [15] and resources dedicated to reducing low-value care [23], the findings of this study have implications for current and future initiatives that seek to reduce low-value care.

Arguably, the most important interaction in healthcare is that between the patient and the clinician. Therefore, it is not surprising that this was the most commonly described context for engaging the public in reducing low-value care. Given that patient demand is a frequently cited barrier to reducing low-value care [15, 43–45], tools that inform patients and their caregivers at the point of care about the lack of utility of certain tests or treatments are promising. Commonly cited tools in current literature include educational materials, shared decision-making, and decision aids. The two studies that evaluated Choosing Wisely educational materials found them to improve general awareness and promote conversations about low-value practices [46, 47]. Comparatively, shared decision-making and decision aids, whose purpose is to guide a choice, are predicted to have a greater effect on changing practice [48]. In shared decision-making, patients and clinicians have a focused, detailed discussion pertinent to the low-value practice in question, thereby enabling patients to develop fully informed preferences [49]. Engaging in decision-making with clinicians can help foster a more trusting relationship, which in turn helps patients accept evidence-based recommendations and improve communication with clinicians [32, 35, 50]. We found that many of the studies that evaluated decision-making tools generally reported an associated reduction in use of the targeted low-value practice. Moreover, the small group of studies that examined the perspectives of patients, caregivers, and clinicians regarding the use of these tools reported that they support their use. Yet, shared decision-making is underused in clinical practice [51–53], likely due to the time and resources required in order for it to be effective [54]. Embracing shared decision-making as a strategy for reducing low-value care will require appropriate infrastructure within the healthcare system and cultural shift among patients and clinicians; however, as highlighted in a recent commentary on the complexities of de-implementation [15], taking such measures to address patient-level factors that affect de-implementation will be the key to the success of future de-implementation initiatives.

Our study identified two additional areas for public involvement in reducing low-value care—policy/administrative decision-making and research. At the policy level, members of the public have most commonly been involved in prioritizing low-value practices through community juries and citizen’s councils. For example, Australia has hosted community juries to examine the public’s perception about disinvestment for assisted reproductive technologies [55] and folate pathology testing [56]. The rationale for involving the public at this level is to gain their insight and perspectives to supplement those of administrators and policy makers [57, 58]. However, in contrast to the patient-clinician interaction where stakeholders unanimously agreed on the value of patient-targeted tools to reduce low-value care, stakeholders are uncertain about whether members of the public should be involved in policy-making and healthcare administration decisions surrounding low-value care. Recognizing that opportunities for public involvement in policy may be shaped by the country, level of government or institution, and nature of the low-value practice in question, this is an aspect of reducing low-value care that requires additional research. Within low-value care research, the public has contributed to the assessment of barriers and facilitators to reducing low-value care, developing and testing tools and interventions for reducing low-value care, and evaluating outcomes of interventions to reduce low-value care through the reporting of important patient-centred outcomes. These studies have acknowledged that involving the public in research can bring meaningful insight and increase the effectiveness of patient-targeted tools and interventions [59, 60]. However, since few studies explain the rationale for or influence of public engagement in low-value care research, it is challenging to make conclusions about the impact of this involvement. Additionally, we did not identify any studies that examined stakeholder perspectives about involving patients as partners in low-value care research. Research that aims to explore how to successfully reduce low-value care will inform the implementation of initiatives at the administrative and policy level, which have the potential to create change on the largest scale. For this reason, understanding how to effectively engage patients and the public early on in the research process is imperative to the development of successful initiatives to reduce low-value care.

The findings of this scoping review must be interpreted within the context of its limitations. First, it is possible that in spite of being peer reviewed and rigorously developed by medical librarians, our electronic database searches may have missed relevant citations. This is potentially due to (1) restriction of the search to the English language and (2) lack of Medical Subject Heading (MeSH) terms to identify low-value care literature that forced the use of a large number of key synonyms and related terms. Restriction to the English language was done because much of the terminology pertaining to low-value care (e.g., low-value, overuse, and de-implementation) is language-specific and may not translate well across languages. Low-value care synonyms and related terms derived from other contemporary literature reviews provided a comprehensive list of terms to include in the electronic database search [27]. It is possible that in combination with terms used to capture “public” and “public involvement,” our search obtained a disproportionate number of citations focused on the patient-clinician interaction, with fewer citations focused on public involvement within policy and research settings. However, given that our final review included 218 citations, of which 31 and 56 focused on policy and research contexts, respectively, it is unlikely that our decision to restrict the search to the English language literature and search term selection missed a sufficiently large number of citations so as to alter our main results. Future evidence syntheses could use our work as a launching point to focus on public involvement in reducing low-value care within the policy and research contexts. Second, mapping included studies to our conceptual framework was a potentially subjective process at risk for misclassification bias. To minimize this risk, all studies were classified in duplicate, agreement checked, and disagreements resolved by consensus or consultation with a third reviewer. Finally, as this was a scoping review, we did not assess the quality of included articles. As described in the recently published PRISMA extension for scoping reviews, article quality assessment is not a typical feature of scoping reviews unless it aligns with the objectives of the review and is practical to complete [25]. The number and heterogeneity of included citations precluded any meaningful assessment of quality of included articles, nor would such data have materially changed the main results of the study.

## Conclusions

In conclusion, there is a large body of literature examining public involvement in reducing low-value care. Current literature suggests that patients and caregivers should be engaged in initiatives to reduce low-value care through point-of-care strategies that include patient-targeted educational materials and shared decision-making tools. As shared decision-making is currently reported to be underused in clinical practice, use of shared decision-making to facilitate de-implementation of low-value care is likely to require additional infrastructure within the healthcare system and a cultural shift among patients and clinicians. In contrast, the perceived utility of public involvement in policy-making and healthcare administration regarding low-value care was questioned by both the public and healthcare administrators. Thus, there is a need to further understand the public’s role in these contexts. As initiatives to reduce low-value care evolve, additional research should examine stakeholder perspectives and quantify the impact of public involvement on reducing low-value care.

## Supplementary information

**Additional file 1.** MEDLINE search strategy. Complete search strategy used for MEDLINE database.

**Additional file 2.** Information sources accessed through the Canadian Agency for Drugs and Technologies in Health (CADTH) Grey Literature Search Tool. List of relevan data sources accessed through the CADTH tool.

**Additional file 3. **Characteristics of citations included in the review (*n* = 218). Bibliographic table of included studies.

## Data Availability

All data generated or analyzed during this study are included in this published article and its supplementary information files.

## Abbreviations

CADTH: Canadian Agency for Drugs and Technologies in Health
NHS: National Health Service
NICE: National Institute for Health and Care Excellence
PRESS: Peer Review of Electronic Search Strategies
PRISMA-ScR: Preferred Reporting Items for Systematic Reviews and Meta-analyses extension for Scoping Reviews
