# Supplementary material for: Understanding the public’s role in reducing low-value care: a scoping review
Source: Implement Sci. 2020 Apr 7;15:20. doi: 10.1186/s13012-020-00986-0 (PMC7137456; doi:10.1186/s13012-020-00986-0)
Supplement: Supplementary file 1 — Additional file 1. MEDLINE search strategy. Complete search strategy used for MEDLINE database. [file 13012_2020_986_MOESM1_ESM.docx]

**Additional File 1.** MEDLINE search strategy.

| **#** | **Searches** |
| --- | --- |
| 1 | health services misuse/ or medical overuse/ |
| 2 | Unnecessary Procedures/ |
| 3 | ((misuse* or overuse* or unnecessary or ineffective or overtreat* or overdiagnos* or overutilis* or overutiliz* or low value or waste*) adj5 (health or healthcare or care or procedure* or intervention* or test* or treatment*)).tw,kf. |
| 4 | ((abandon* or contradict* or refute* or refuting or reassess* or re-assess* or obsole* or revers* or delist* or de-list* or disinvest* or dis-invest* or discontinu* or dis-continu* or decommission* or de-commission* or deadopt* or de-adopt* or de-implement* or deimplement*) adj5 (medical or health or healthcare or policy or procedure* or intervention*)).tw,kf. |
| 5 | 1 or 2 or 3 or 4 |
| 6 | patient participation/ or community participation/ |
| 7 | patient satisfaction/ or patient preference/ |
| 8 | ((patient* or family* or families or public or citizen* or consumer*) adj5 (perception* or engag* or involv* or participat* or decision* or interaction* or role* or aware* or conversation* or responsibilit* or discuss*)).tw,kf. |
| 9 | 6 or 7 or 8 |
| 10 | 5 and 9 |
| 11 | choosing wisely.mp. |
| 12 | 10 or 11 |
| 13 | Limit 12 to English language |
