# Supplementary material for: Understanding the public’s role in reducing low-value care: a scoping review
Source: Implement Sci. 2020 Apr 7;15:20. doi: 10.1186/s13012-020-00986-0 (PMC7137456; doi:10.1186/s13012-020-00986-0)
Supplement: Supplementary file 2 — Additional file 2. Information sources accessed through the Canadian Agency for Drugs and Technologies in Health (CADTH) Grey Literature Search Tool. List of relevan data sources accessed through the CADTH tool. [file 13012_2020_986_MOESM2_ESM.docx]

**Additional File 2.** Information sources accessed through the Canadian Agency for Drugs and Technologies in Health (CADTH) Grey Literature Search Tool.

| **Information Source** | **Website** |
| --- | --- |
| The Alberta College of Family Physicians (ACFP). Tools for Practice | <http://acfp.ca/WhatWeDo/ToolsforPractice.aspx> |
| Canadian Agency for Drugs and Technologies in Health (CADTH) | <https://www.cadth.ca/search?keywords> |
| [Health Quality Council of Alberta](http://www.hqca.ca/about) (HQCA). Completed Reviews | <http://hqca.ca/studies-and-reviews/completed-reviews/> |
| Health Quality Ontario (HQO). Publications and OHTAC Recommendations. | <https://www.hqontario.ca/evidence/publications-and-ohtac-recommendations> |
| Ottawa Hospital Research Institute (OHRI). Knowledge Synthesis Group | <http://www.ohri.ca/ksgroup/publications.asp> |
| University of British Columbia. Centre for Health Services and Policy Research | <http://chspr.ubc.ca/pubs/pub-search> |
| INAHTA Secretariat. International Network of Agencies for Health Technology Assessment (INAHTA) | <http://www.inahta.org/publications> |
| [Australian Government Department of Health and Ageing. Medical Services Advisory Committee](http://www.msac.gov.au/internet/msac/publishing.nsf/Content/about-us-lp-1) (MSAC). Completed Assessments and Reviews | http://www.msac.gov.au/internet/msac/publishing.nsf/Content/completed-assessments |
| [Monash Health. Centre for Clinical Effectiveness](http://www.monashhealth.org/page/About_Us/) (CCE). Current Evidence Reviews | <http://www.monashhealth.org/page/Current> |
| [Institute of Technology Assessment](http://www.oeaw.ac.at/ita/en/about-us) (ITA). | <http://www.oeaw.ac.at/ita/en/projects> |
| [Kenniscentrum voor de Gezondheidszorg / Le Centre d'expertise des soins de santé](https://kce.fgov.be/about-kce). Belgian Health Care Knowledge Centre (KCE) | [https://kce.fgov.be/search/apachesolr_search/?filters=type:biblio%20ss_biblio_secondary_title:"KCE%20Reports"&retain-filters=1](https://kce.fgov.be/search/apachesolr_search/?filters=type:biblio%20ss_biblio_secondary_title:%22KCE%20Reports%22&retain-filters=1) |
| [Health Information and Quality Authority](http://www.hiqa.ie/about-us). Health Technology Assessments | [http://www.hiqa.ie/healthcare/health-technology- assessment/assessments](http://www.hiqa.ie/healthcare/health-technology-%09assessment/assessments) |
| [Swedish Council on Health Technology Assessment](http://www.sbu.se/en/About-SBU) (SBU). | <http://www.sbu.se/en/> |
| Healthcare Improvement Scotland | <http://www.healthcareimprovementscotland.org> |
| [National Institute for Health and Care Excellence](http://www.nice.org.uk/aboutnice/) (NICE). | <http://www.nice.org.uk/> |
| [National Institute for Health and Care Excellence](http://www.nice.org.uk/aboutnice/) (NICE). Advice List. Published evidence summaries | <http://www.nice.org.uk/advice?type=esnm> |
| National Health Service UK (NHS). NHS England | <http://www.england.nhs.uk/> |
| Agency for Healthcare Research and Quality | <https://effectivehealthcare.ahrq.gov/index.cfm/search-for-guides-reviews-and-reports/> |
| [Blue Cross and Blue Shield Association](http://www.bcbs.com/blueresources/tec/)**.** Technology Evaluation Center (TEC) | <http://www.bcbs.com/blueresources/tec/topic.html> |
| California Technology Assessment Forum (CTAF). Assessments | <http://www.ctaf.org/reports> |
| Institute for Clinical and Economic Review (ICER). | <http://www.icer-review.org/index.php/Table/Appraisals> |
| Washington State Health Care Authority (HCA). Health Technology Assessment Findings | <http://www.hca.wa.gov/hta/Pages/Forms/HTA_Findigns.aspx> |
| Toronto Health Economics and Technology Assessment Collaborative (THETA). THETA Publications and Knowledge Translation to Policy (KT) Activities | <http://theta.utoronto.ca/content.php?pid=41861&sid=3372336> |
| Alberta Medical Association. Towards Optimized Practice (TOP) | <http://www.topalbertadoctors.org/cpgs.php?sid=1> |
| Winnipeg Regional Health Authority (WRHA). Evidence Informed Practice Tools | <http://www.wrha.mb.ca/professionals/ebpt> |
| <http://www.bpac.org.nz/Default.aspx> | <http://www.bpac.org.nz/Default.aspx> |
| McMaster University, McMaster Health Forum. Health Systems Evidence | <http://www.healthsystemsevidence.org> |
| Google | <http://www.google.com> |
| Google Scholar | <http://scholar.google.ca> |
| Canadian Agency for Drugs and Technologies in Health (CADTH). Rx for Change. | <https://www.cadth.ca/resources/rx-for-change/database/browse> |
| Canadian Nurses Association (CAN). Download/Buy | <https://www.cna-aiic.ca/en/download-buy> |
| Registered Nurses’ Association of Ontario (RNAO). Publications & Resources | <http://rnao.ca/resources> |
