# Supplementary material for: Understanding the public’s role in reducing low-value care: a scoping review
Source: Implement Sci. 2020 Apr 7;15:20. doi: 10.1186/s13012-020-00986-0 (PMC7137456; doi:10.1186/s13012-020-00986-0)
Supplement: Supplementary file 3 — Additional file 3. Characteristics of citations included in the review (n = 218). Bibliographic table of included studies. [file 13012_2020_986_MOESM3_ESM.docx]

**Additional File 3.** Characteristics of citations included in the review (n=219)

| **Author** | **Year** | **Country** | **Study Design** | **Low-value practice** | **Clinical setting** | **Strategy for public involvement** |
| --- | --- | --- | --- | --- | --- | --- |
| **Studies that aimed to examine stakeholder perspectives about how the public should be involved in reducing low-value care** | | | | | | |
| ***Level of public involvement: Patient-clinician interaction*** | | | | | | |
| Ellen | 2018 | Israel | Qualitative interviews/focus groups | Low-value care in general | Outpatient clinic | Patients need to be educated on the dangers of overuse |
| Embrett | 2018 | Canada | Qualitative interviews/focus groups | Low-value care in general | Not specified | Patients should be engaged in conversations surrounding low-value care during the clinical encounter |
| Kanzaria(1) | 2015 | United States of America | Cross-sectional | Advanced diagnostic imaging | Emergency Department | Emergency physicians perceived education of patient and families and involving patients in shared decision making for diagnostic testing for low probability outcomes. |
| Kullgren(2) | 2018 | United States of America (USA) | Qualitative interviews/focus groups | Medications (other than metformin) to achieve a hemoglobin A1c below 7.5%, benzodiazepines or other sedative-hypnotics for insomnia, agitation or delirium, routine PSA-based screening for prostate cancer | Primary Care | Patients want personalized decisions that are informed by the benefits and potential risks about following recommendations to reduce low value-care |
| Linsky(3) | 2014 | United States of America (USA) | Qualitative interviews/focus groups | Unnecessary medications | Primary Care | Patients are willing to discontinue unnecessary medications but stressed the importance of having a strong patient-provider relationship and engaging in shared decision making |
| Rohrbacher(4) | 2007 | Germany | Cross-sectional | Low-value care in general | Emergency Department | Patients want to discuss their preferences and personal experiences with their physician to arrive at the most evidence-based decision |
| Scales(5) | 2017 | United States of America (USA) | Cross-sectional | Unnecessary antibiotics in nursing homes | Long term care | Educating the nursing home residents and their families about why it is unnecessary to prescribe antibiotics |
| Schoenborn(6) | 2017 | United States of America (USA) | Qualitative interviews/focus groups | Cancer screening when life expectancy is limited | Outpatient screening | Interviewed patients about how they want to be involved in discussing stopping cancer screening - patients value a trusting relationship with their physician and want to incorporate health status into the discussion about screening cessation |
| ***Level of public involvement: Healthcare administration and policy*** | | | | | | |
| Daniels(7) | 2018 | United Kingdom (UK) | Mixed methods | Low-value care in general | Not specified | Involving citizens in disinvestment decisions |
| Hislop(8) | 2011 | United Kingdom (UK) | Qualitative interviews/focus groups | Low-value care in general | Not specified | Taxpayers lack required knowledge and patients lack required impartiality to be heavily involved in national policy-making on disinvestment |
| **Studies that described or tested a strategy for involving the public in reducing low-value care** | | | | | | |
| ***Level of public involvement: Patient-clinician interaction*** | | | | | | |
| Adisso | 2018 | Canada | Community outreach/education | Unnecessary antibiotics | Not specified | Program in a public library to increase public awareness and knowledge about shared decision-making and antibiotics use for ARTI;  Post-workshop survey collated suggestions and improvements from participants |
| Aggarwal(9) | 2014 | United Kingdom | Narrative review | Low-value cancer treatment including imaging, radiation, drugs | Hospital | User charges to nudge patients away from low-value services |
| Al Kharji | 2018 | Canada | Narrative review | Unnecessary fluoroscopy-guided procedures | Hospital | Patients should be educated and informed about the risks of the procedures |
| Alber(10) | 2017 | Germany | Qualitative analysis | Low-value care in general | Primary care | Educating patients on evidence, advantages of a wait and see approach, importance of health-conscious behaviour, price/cost transparency |
| Allam(11) | 2015 | Switzerland | Randomized controlled trial | Health care over utilization in general and prescription medication overuse for patients with rheumatoid arthritis | Not specified | Online social support to increase knowledge and empower rheumatoid arthritis patients, improve their health care utilization and reduce medication overuse |
|  |  |  |  |  |  |  |
| Almoosa(12) | 2010 | United States of America | Quality Improvement | Low-value care in general | Hospital | Multidisciplinary family meetings to discuss patient's prognosis, family expectations and develop a care plan with the goal of reducing the use of futile treatments prior to death |
| Anstey(13) | 2014 | United States of America | Framework | Low-value care in general | Hospital | Treatment decisions should take patients' preferences into account to avoid overutilization of care the patient does not desire |
| Arterburn | 2012 | United States of America | Before-and-after | Unnecessary surgery for knee and hip osteoarthritis | Hospital | Patient decision aids in DVD and booklet format via US mail, available for order in the EMR. Alternatively, patients could view the decision aids online on Group Health’s secure website for members. |
| Ashe(14) | 2006 | United States of America | Case-control | Antibiotics | Primary Care | Public education in the form of a waiting room poster |
| Atkinson | 2018 | Canada | Editorial/commentary/little-to-the-editor | Low-value care in general | Emergency Department | Patients should engage with patient-focused posters and educational phamplets that explain in lay terms the individual harms or opportunity costs of unnecessary care |
| Bansback(15) | 2016 | Canada | Cohort | Imaging tests for low back pain | Emergency Department | Choosing Wisely pamphlet to improve knowledge and intentions surrounding imaging tests for low back pain |
| Bauchner | 2001 | United States of America | RCT | Inappropriate oral antibiotics | Primary care | Video to educate parents about antibiotic resistance and appropriate antibiotic use; also a brochure with similar information |
| Betran | 2018 | Switzerland | Narrative review | Unnecessary caesarean sections | Hospital | Patients can use educational materials and personalized decision aids to help inform them of the risks of unnecessary caesearn sections |
| Bezin(16) | 2015 | France | Cohort | Statins | Not specified | Public discussion and media coverage on appropriate use of statins |
| Bobbio | 2019 | Italy | Narrative review | Low-value care in general | Not specified | Choosing Wisely and Altroconsumo created documents for patients and the public to help them identify and be aware of low-value practices. An app was developed containing recommendations and documents for the general public |
| Bodenheimer(17) | 2005 | United States of America | Narrative review | Low-value care in general | Not specified | Shared decision making to help discuss available options and their consequences, and bring evidence based knowledge to the point of care |
| Bogler | 2019 | Canada | Editorial/commentary/little-to-the-editor | Polypharmacy | Not specified | Patients and caregivers should engage in shared decision-making with their physician about what medications are actualy safe and necessary |
| Bolt(18) | 2016 | Netherlands | Mixed methods | Low-value care in general | Not specified | Assessed patient and family perceptions of inappropriate care at the end of life, including over treatment or prolonging unnecessary treatment |
| Borasio(19) | 2016 | Switzerland | Narrative review | Low-value care in general | Hospital | Advanced care planning to discuss patient goals and preferences to avoid inappropriate or unnecessary procedures at the end of life |
| Born(20) | 2017 | Canada | Editorial/commentary/letter-to-the-editor | Low-value care in general | Not specified | Involving patient and public representatives to incorporate their perspectives into Choosing Wisely recommendations; Media campaigns providing the public with information about overuse; Shared-decision making with providers to make informed choices about their treatment plans |
| Born | 2019 | Canada | Editorial/commentary/litter-to-the-editor | Low-value care in general | Not specified | Choosing Wisely campaign aims to educate patients about low-value care and promote shared decision-making between patients and physicians to reduce low-value care |
| Bozzella | 2018 | United States of America | Narrative review | Inappropriate antibiotic use | Outpatient clinic | Educate patients, families, and the general public about when antibiotics are and are not necessary and about the risks of using them unnecessarily |
| Brownlee(21) | 2016 | United States of America | Editorial/commentary/letter-to-the-editor | Low-value care in general | Not specified | Use of shared-decision making tools, advanced care planning and public involvement in decision making and deliberation as a part of health care cost control and planning |
| Buist(22) | 2016 | United States of America | Cross-sectional | Low-value care in general | Primary Care | Specific handouts to give to patients explaining why a certain test is or is not indicated and the risks and benefits; Public awareness campaign to raise the profile of the importance of reducing low-value care |
| Burgess(23) | 1989 | Canada | Narrative review | Low-value care in general | Not specified | Patients should be educated about the costs, risks and benefits of medical treatments and should participate in medical decisions |
| Burns(24) | 2014 | United States of America | Narrative review | Low-value care in general | Not specified | Create financial disincentives for commonly overused services |
| Burns | 2019 | United States of America | Consensus method | Unnecessary blood transfusions | Hospital | Patients and physicians should engage in patient-centered decision making to discuss risks of transfusions and alternative options |
| Chernew(25) | 2007 | United States of America | Editorial/commentary/letter-to-the-editor | Low-value care in general | Not specified | Patients take on more cost sharing for low-value services |
| Chien | 2019 | United States of America | Narrative review | Low-value care in general | Not specified | Engage patients in shared decision making, tailor care to the individual needs of patients, and assist them more effectively in self-monitoring. For vulnerable populations, multifaceted interventions that are sensitive and culturally appropriate to increase engagement with patients and families about low value care |
| Choosing Wisely Canada | 2018 | Canada | Editorial/commentary/litter-to-the-editor | Antibiotics for upper respiratory tract infections | Primary care | Patients can use visual and interactive aids to understand the implications of using antibiotics unnecessarily and can engage in shared decision-making with their provider to make a decision about their care |
| Choosing Wisely Canada | 2019 | Canada | Editorial/commentary/litter-to-the-editor | Pap tests for women younger than 21 or older than 69 | Primary care | Patients can use visual and interactive aids to understand the implications of using antibiotics unnecessarily and can engage in shared decision-making with their provider to make a decision about their care |
| Choosing Wisely Canada | 2018 | Canada | Editorial/commentary/litter-to-the-editor | Antibiotics for upper respiratory tract infections | Primary care | Patients can use visual and interactive aids to understand the implications of using antibiotics unnecessarily and can engage in shared decision-making with their provider to make a decision about their care |
| Colla(26) | 2017 | United States of America | Systematic review | Low-value care in general | Not specified | Cost-sharing to shift cost of low-value care towards patients; direct patient education to inform them about low-value care; shared decision making; provider report cards so that they choose a high value provider |
| Colla(27) | 2014 | United States of America | Editorial/commentary/letter-to-the-editor | Low-value care in general | Not specified | Patient education to support patients in making informed decision based on service value through structured shared decision making/decision aids; patient cost sharing or value based insurance design |
| Cooper(28) | 2015 | United States of America | Framework | Low-value care in general | Hospital | Shared decision making to prevent overuse in surgeries |
| Dollman(29) | 2005 | Australia | Before-and-after | Antibiotic use for upper respiratory tract infections, sinusitis and otitis media | Primary Care | Pamphlets highlighting risks and benefits of antibiotic use for URTIs distributed to general practices, pharmacies, hospitals, schools, childcare centres and clubs; articles in the local community paper |
| Dowling | 2019 | Canada | Cross-sectional | CT scans for minor traumatic brain injury | Emergency Department | Patient infographic on perceptions of the risks and benefits of a CT scan for minor traumatic brain injury;  Survey evaluated baseline patient perceptions of when a CT scan is necessary, risks associated with CTs, and patient willingness to engage in a discussion with their physician about the risks and benefits of medical imaging |
| Eddy | 2018 | New Zealand | Editorial/commentary/letter-to-the-editor | Low-value care in general | Hospital | Patients should using the four Choosing Wisely questions to raise discussion with their healthcare provider when test or procedures are being recommended. There is also a consumer information campaign, which provides background material relating to each recommendation, to support informed decision making and discussions |
| Ellen | 2018 | Canada | Qualitative analysis | Low-value care in general | Not specified | Foster better communication and shared decision-making between providers and patients, educate patients/citizens about what health services they need, develop mass-media campaigns to raise awareness about the need to address overuse |
| Engineer(30) | 2018 | United States of America | Pilot study | Computed tomography for mild head injury in the Emergency Department | Emergency Department | A clinical decision support tool that involved a structured discussion between providers and patients |
| Fendrick(31) | 2010 | United States of America | Editorial/commentary/letter-to-the-editor | Low-value care in general | Not specified | Patients take on more of the cost sharing for low-value services |
| Filipetto(32) | 2008 | United States of America | Cross-sectional | Antibiotic use for respiratory and common colds | Primary Care | Determine patient knowledge and perception of upper respiratory tract infections and appropriateness of antibiotic treatment; Assess patient satisfaction on physician decision to prescribe/not prescribe antibiotics |
| Francis(33) | 2009 | United Kingdom | Randomized controlled trial | Antibiotics | Primary care | Intervention included an interactive booklet on respiratory tract infections, designed to be used within the consultation and then provided to patients as a take home resource; Assess parent satisfaction, level of reassurance, parental enablement or parents rating of usefulness of information received in the consultation in intervention and control |
| Fraser(34) | 1997 | Canada | Randomized controlled trial | Unnecessary caesarean section | Hospital | Prenatal education and support program |
| Frush(35) | 2014 | United States of America | Narrative review | Computed tomography in the Emergency Department | Emergency Department | Engage families (and the patient if they are old enough) in decisions regarding the use of computed tomography for evaluation of paediatric patients in the Emergency Department |
| Ghanouni(36) | 2016 | United Kingdom | Cross-sectional | Low-value care in general | Not specified | Determine how the public conceptualizes the term over diagnosis as a result of potentially unnecessary screening tests |
| Gonzales(37) | 2008 | United States of America | Non-randomized controlled trial | Antibiotics | Primary Care | Mass media campaign (paid outdoor advertising, earned media, and physician advocacy) to educate the public on the misuse of antibiotics |
| Green(38) | 2018 | United States of America | Qualitative interviews/focus groups | Low-value care in general | Not specified | Assessed patient awareness of overuse; Patients suggested that overuse may be reduced when the patient is involved in shared decision making |
| Gupta | 2018 | United States of America | Non-randomized controlled trial | Inpatient EPOCH-based chemotherapy regimens | Hospital | Patient targeted educational materials;  Patient satisfaction was evaluated as part of the de-implementation intervention |
| Hemo(39) | 2009 | Israel | Cross-sectional | Antibiotic use for upper respiratory tract infection | Not specified | Media campaign to reduce antibiotic overuse among children |
| Hersch(40) | 2015 | Australia | Randomized controlled trial | Breast cancer screening | Outpatient screening | Decision aid comprising evidence-based explanatory and quantitative information on over-detection, breast cancer mortality reduction, and false positives |
| Herwig(41) | 2017 | Germany | Qualitative interviews/focus groups | Cardiac catheterization | Hospital | Study will explore the patient's perspective on the overuse of cardiac catheterization |
| Hess(42) | 2012 | United States of America | Randomized controlled trial | Cardiac stress testing in patients at low risk for acute coronary syndrome | Emergency Department | Decision aid about cardiac stress testing to improve patient knowledge and engage in shared decision making with their care provide |
| Hess(43) | 2016 | United States of American | Randomized controlled trial | Cardiac stress testing in patients at low risk for acute coronary syndrome | Emergency Department | Decision aid about cardiac stress testing to improve patient knowledge and engage in shared decision making with their care provide |
| Hiscock | 2018 | Australia | Systematic review | Unnecessary imaging and pathology testing in pediatric populations | Not specified | Family education materials; group education classes/seminars, behavioral therapy (sessions using education, reassurance, dietary intervention, exercise instruction, and guided imagery); patient/family education and information on variations of the clinical pathway |
| Hultman | 2019 | United States of America | Editorial/commentary/little-to-the-editor | Low-value care in general | Outpatient clinic | Crowdsourced websites for specific conditions as an online forum. Education can utilize video presentations that can be augmented by printed educational materials to be taken home |
| Jerardi | 2013 | United States of America | Quality improvement | Voiding cystourethrogram in children with first UTI with normal renal and bladder ultrasound | Hospital | Discharge educational materials and information sheets for families would automatically become available in the EMR for eligible patients. Additional online educational materials were available to families, patients, and community providers on the hospital Web site |
| Jessup | 2018 | Australia | Narrative review | Low-value care in general | Not specified | Patients should be educated about the risks and benefits of medical tests and treatments, avoiding the use of medical jargon and using visual aids to help them undersand probabilistic information |
|  |  |  |  |  |  |  |
| Kanzaria(44) | 2015 | United States of America | Cross-sectional | Low-value care in general | Emergency Department | Patients and physicians should engage in shared decision making as a solution to reduce over-testing |
| Keating(45) | 2018 | United States of America | Editorial/commentary/letter-to-the-editor | Routine mammography in women under 50 years of age | Outpatient screening | Patient/physician shared-decision making to reduce unnecessary breast cancer screening |
| Khunpradit(46) | 2011 | Thailand | Systematic review | Unnecessary caesarean section | Hospital | Education/support programs; decision aids |
| Kinkade(47) | 2016 | United States of America | Narrative review | Antibiotics for cough | Primary Care | Educating patients on natural course of disease and unnecessary antibiotic use |
| Kline(48) | 2017 | United States of America | Cross-sectional | Nuclear myocardial perfusion imaging (MPI) | Outpatient clinic | Assessed patient knowledge of the value of MPI testing, including when it is necessary, the costs, and risks |
| Kotwal(49) | 2017 | United States of America | Narrative review | Breast, colorectal, lung, and prostate screening in the elderly | Outpatient screening | Shared-decision making incorporating guidelines, patient preferences and patient life expectancy estimates |
| Kozhimannil(50) | 2013 | United States of America | Cohort | Caesarean section | Hospital | Patient-centered decision making, providing them with the full information on risks, benefits and alternatives associated with medical care at the time of childbirth |
| Kronemyer | 2018 | United States of America | Editorial/commentary/letter-to-the-editor | Low-value care in general | Not specified | Focused patient education can lead to improved patient knowledge and, in turn, increased shared decision making. Decision aids are a method to accomplish this |
| Kuehn(51) | 2012 | United States of America | Website item/news | Low-value care in general | Not specified | American Board of Internal Medicine Foundation partnership with Choosing Wisely Campaign to disseminate materials to public in Consumer Reports publication |
| Kullgren | 2019 | United States of America | Quality improvement | Overtreatment of diabetes, insomnia and anxiety | Primary care | Patients were mailed point-of-care patient handouts explaining the low-value treatments prior to their schedule primary care visit |
| Larson(52) | 2010 | United States of America | Editorial/commentary/letter-to-the-editor | Imaging procedures, image-guided therapy, radiation therapy | Not specified | Patients need to be made aware of the appropriateness of their care and use this information in their decisions |
| Laws(53) | 2012 | United States of America | Editorial/commentary/letter-to-the-editor | Low-value care in general | Not specified | Patient-physician discussion about avoiding inappropriate care so that patients understand that some interventions may not be necessary for their conditions and circumstances |
| Legare(54) | 2012 | Canada | Randomized controlled trial | Antibiotics for respiratory tract infections | Primary care | Shared-decision making between patient and physicians (trained in shared decision making) to discuss antibiotics; Patients evaluated decisional conflict, perception that shared decision making occurred, quality of decision made, adherence to the decision, decisional regret, repeat consultation, quality of life, and intention to participate in shared decision making in future consultations regarding the use of antibiotics |
| Legemate(55) | 2016 | Netherlands | Editorial/commentary/letter-to-the-editor | Low-value care in general | Hospital | Patients and physicians should discuss the risks associated with a procedure and the number of patients who would not benefit from the treatment to help discourage patients from choosing low-value treatments |
| Leung(56) | 2017 | Japan | Health Education/Public Outreach | Antibiotics | Community pharmacy | Public competition to create videos for education on antibiotic misuse and dissemination of the video in public schools |
| Levinson(57) | 2014 | Canada | Editorial/commentary/letter-to-the-editor | Low-value care in general | Not specified | Physicians and patients should engage in conversations about unnecessary tests, treatments, and procedures to help patients make effective choices; Educational efforts targeted to patients and the public are required in order to engage them in the dialogue |
| Levinson(58) | 2014 | Canada | Narrative review | Low-value care in general | Not specified | Choosing Wisely Campaign encourages patients to discuss low-value care with their care providers and prevent harm; Patient surveys about attitudes towards low-value care and care experiences |
| Litvin | 2018 | United States of America | Qualitative interviews/focus groups | PSA screening for prostate cancer | Outpatient screening | Patient education videos or materials to convey the benefits and harms of screening and face-to-face shared decision-making conversations |
| Macfarlane(59) | 2002 | United Kingdom | Randomized controlled trial | Antibiotics for acute bronchitis | Primary Care | Information leaflet on why antibiotics aren't necessary |
| Maire(60) | 2014 | United States of America | Editorial/commentary/letter-to-the-editor | Low-value care in general | Not specified | Patients and physicians should use Choosing Wisely recommendations to make effective and safe decisions on the most appropriate care based on their specific situation |
| Martin(61) | 2004 | United States of America | Cross-sectional | Antibiotic use in rhinosinusitis | Primary Care | Assessed patient exceptions about receiving an antibiotic prescription and how they would feel about getting a back-up prescription as a method to reduce antibiotic use |
| Mason(62) | 2015 | United States of America | Website item/news | Low-value care in general | Not specified | Patients would have to pay for low-value services, particularly those that may cause harm |
| McCaffery(63) | 2016 | Australia | Editorial/commentary/letter-to-the-editor | Low-value care in general | Not specified | Mass media and direct to consumer campaigns about low-value practices; shared decision making/patient decision aids; community juries to discuss what services are available or reimbursed by health funds |
| McCanne(64) | 2018 | United States of America | Website item/news | Low-value care in general | Not specified | Increasing cost-sharing for low-value services to reduce their use |
| Montgomery(65) | 2007 | United Kingdom | Randomized controlled trial | Unnecessary caesarean section | Hospital | Decision aid for patient use; Evaluated patient satisfaction and anxiety with the process |
| Moore(66) | 2016 | United States of America | Qualitative interviews/focus groups | Overuse of elective labor induction | Hospital | Shared-decision making to help women make value and preference based decisions to reduce overuse in maternity care |
| Morgan(67) | 2017 | United States of America | Systematic review | Low-value care in general | Not specified | Shared decision making using a decision aid to reduce cardiac testing for low-risk chest pain |
| Morgan(68) | 2017 | United States of America | Framework | Low-value care in general | Not specified | Culture of healthcare consumption may be influenced through public education (eg, Choosing Wisely patient resources) and public health campaigns; In patient-clinician interactions, shared decision making and continuity of care likely reduce overuse |
| Morgan(69) | 2002 | United Kingdom | Before-and-after | Long term use of benzodiazepines | Primary Care | A patient letter explaining the problems association with long term benzodiazepine use and encouraging patients to gradually reduce their intake, and contact their GP for discussion if desired |
| Moynihan(70) | 2015 | Australia | Cross-sectional | Screening | Not specified | Assessed knowledge of overdiagnosis as a result of low-value screening: most adults have not been informed about over diagnosis attached to screening tests, community members expressed desire for routine provision of benefits and risk of overdiagnosis |
| Murphy(71) | 2015 | United Kingdom | Editorial/commentary/letter-to-the-editor | Blood transfusions | Hospital | Education of patients, for whom transfusion may be a treatment option, about individualized blood management and blood avoidance should be an integral part of relevant care pathways; Recommendations for blood management include informing patient of the risks and benefits of blood transfusion and documenting this discussion in the medical record |
| Nagler(72) | 2017 | United States of America | Cross-sectional | Mammogram | Outpatient screening | Assess women's awareness of overdiagnosis and overtreatment related to breast cancer screening |
| Navaee | 2015 | Iran | RCT | Voluntary caesarean births in primiparous women | Hospital | A 90-min lecture and role-playing session about the advantages and disadvantages of normal delivery vs ceasarean |
| Newton(73) | 2017 | United States of America | Narrative review | Overuse in emergency medicine | Emergency Department | Shared decision making has been suggested as a strategy to reduce low value care in the Emergency Department and involve patients in their care |
| Nilsen(74) | 2017 | United States of America | Narrative review | Chemotherapy and radiation in head and neck cancer in palliative care patients | Hospital | Patients and families should be involved in shared decision making to grant influence to patients about treatment preferences and values in treatment for head and neck cancer that fits with these preferences; Informing patients of burden and benefits of chemo and radiation therapy for palliative symptoms is important for their choices around care |
| O’Keefe | 2019 | Australia | Editorial/commentary/letter-to-the-editor | Unnecessary imaging, medication, and surgery for back pain | Not specified | Prioritize evidence-based communication on social media platforms; develop apps based on theory and rigorous pre-testing; target online messaging (eg, Google Ads) such that when someone searches ‘back pain’ they would receive appropriate information instead of advertisements about non-evidence-based treatments—as tested with cancer risk and tanning bed use; and promote citizen science endeavors which establish online communities in specific areas to engage the public in research |
| Parmar(75) | 2015 | Canada | Editorial/commentary/letter-to-the-editor | Low-value care in general | Not specified | Educate patients and ensure they are aware of risks of low-value care |
| Pathirana | 2017 | Australia | Narrative review | Low-value care in general | Not specified | Promote shared decision-making; rigorous evaluation of the effects of both new and existing diagnostic technology on health outcomes to re-evaluate the sensitivity of tests |
| Pereko(76) | 2015 | Namibia | Case-control | Antibiotics | Community pharmacy | Assess knowledge, attitudes and behaviour of the general population regarding antibiotic misuse for cold and flu symptoms |
| Perz(77) | 2002 | United States of America | Before-and-after | Antibiotics | Primary Care | Educational materials for parents of young children and the general public on the misuse of antibiotics |
| Polaris | 2014 | United States of America | Narrative review | Low-value care in general | Not specified | Shared decision making approach involves eliciting patient-centered goals and values in order to reach a treatment plan that is consistent with the patient’s own priorities and preferences, and discussing both benefits and potential harms; |
| Prescrire Editorial Staff(78) | 2014 | France | Website item/news | Low-value care in general | Not specified | Involve the patient in determining the harm-benefit balance of the test/procedure |
| Price(79) | 2013 | United Kingdom | Narrative review | Low-value care in general | Hospital | Patient-provider (or surrogate-provider) discussion about preferences, goals, and the futility of the treatment |
| Pugel | 2018 | United States of America | Quality improvement | Complete blood counts (CBCs), electrocardiograms (EKGs) as routine screening tests in physical examination visits, age-inappropriate dual-energy e-ray absorptiometry (DEXA) scans, imaging for uncomplicated headache | Outpatient clinic | The quality improvement initiative included patient-targeted materials produced by Consumer Reports, including exam-room posters, patient education material available in waiting areas and patient education handouts in exam rooms |
| Razmaria(80) | 2015 | United States of America | Website item/news | Low-value care in general | Not specified | Patients should ask physicians questions about tests and treatments: What are the risks? How will this help me? Are there any other options? What would happen if I don't do anything? Asking these questions will help reduce low-value care |
| Reid(81) | 2017 | United States of America | Difference-in-differences | Low-value care in general | Not specified | Consumer directed health plans involve greater cost-sharing for patients, which is intended to encourage more value-conscious choices |
| Rich | 2018 | United Kingdom | Editorial/commentary/little-to-the-editor | Low-value care in general | Not specified | Promoting patient networks can allow them to support one another and can reduce overmedication. Ensure fully informed consent by discussing potential side effects of treatment options, and decision aids and other forms of communication should be used to facilitate this. Educational videos about screening can reduce patient desire. Enhanced discussions at end of life to reduce unnecessary care |
| Santa(82) | 2012 | United States of America | Narrative review | Low-value care in general | Not specified | Choosing Wisely uses Consumer Reports as a tool to communicate 'what not to do' to consumers and assess their knowledge of low-value practices |
| Santhirapala | 2019 | United Kingdom | Narrative review | Low-value care in general | Hospital | Patients and physicians should engage in shared decision-making to reduce low-value care. Consumer Reports has created patient education materials in the US to facilitate discussions |
| Sawaya(83) | 2017 | United States of America | Qualitative interviews/focus groups | Pelvic examinations in asymptomatic, non-pregnant women | Outpatient clinic | Providing patients with a professional society's recommendation advising against the low-value practice |
| Schleifer(84) | 2012 | United States of America | Qualitative interviews/focus groups | Low-value care in general | Not specified | Explore patient attitudes about the overuse of medical interventions |
| Schlesinger(85) | 2017 | United States of America | Mixed methods | Low-value care in general | Not specified | Assessed consumers knowledge and attitude towards low-value care, assessed the impact of the framing of messages from media campaigns (i.e. Choosing Wisely) on their knowledge, attitudes and intentions |
| Schneiderman(86) | 2003 | United States of America | Randomized controlled trial | Non-beneficial life-sustaining treatments in the intensive care setting | Hospital | Ethics consultations to discuss relevant medical factors, values and preferences, other contextual factors and help reduce the use of non-beneficial life-sustaining treatments |
| Scott(87) | 2013 | Australia | Narrative review | Low-value care in general | Not specified | Empower patients to participate in shared decision making to reduce demand for low-value care |
| Shallcross(88) | 2015 | United Kingdom | Editorial/commentary/letter-to-the-editor | Antibiotics | Not specified | Public media campaigns to raise awareness about overuse of antibiotics |
| Sheridan(89) | 2015 | United States of America | Randomized controlled trial | Prostate screening in men ages 50-69, osteoporosis screening in low-risk women ages 50-64, colorectal screening in men and women ages 76-85 | Outpatient screening | Provided one page evidence-based decision support sheets in different formats (words, numbers, narratives, etc) and tested intention to screen before and after reading the decision support sheet |
| Silverstein(90) | 2016 | Canada | Cross-sectional | Annual ECG testing, use of antipsychotic drugs for patients with dementia, use of antibiotics for treatment of sinusitis, imaging for low back pain, sedative-hypnotic use in patients with insomnia | Primary Care | Assessed knowledge, attitudes and behaviours around low-value practices before and after exposure to a Choosing Wisely Canada patient educational brochure |
| Simpson(91) | 2010 | United States of America | Before-and-after | Elective labour induction | Hospital | Educational classes on induction risk |
| Singh(92) | 2017 | United States of America | Pilot study | Head computed tomography for minor head injury | Emergency Department | Clinician and patient facing electronic tool to guide decisions about computed tomography use, promotes conversation around individualized risk and concerns; Measured patient satisfaction with the way information was shared during the encounter and their trust in physician |
| Skolarus | 2018 | United States of America | Mixed methods | Low-value castration for men with prostate cancer | Hospital | De-implementation intervention will involve physician/patient informed decision-making to reduce low-value care. Interviews will be conducted pre-intervention with patients to determine knowledge of treatments for castration and associated harms;  Patients will be interviewed to explore their preferences and perceived barriers to the de-implementation of castration for prostate cancer treatment |
| Spong(93) | 2015 | United States of America | Narrative review | Unnecessary caesarean delivery | Hospital | Educating patients on the risks associated with caesarean delivery and importance of vaginal delivery |
| Stelfox | 2019 | Canada | Editorial/commentary/little-to-the-editor | Low-value care in general | Hospital | Engaging patients through direct education, shared decision-making, and media campaigns |
| Sutkowi-Hemstreet(94) | 2015 | United States of America | Qualitative interviews/focus groups | Overused screening tests (prostate cancer, osteoporosis, colon cancer, cardiovascular disease) | Outpatient screening | Assessed how patients think about the harms and benefits of overused screening tests and how they consider these factors when making decisions |
| Tannenbaum(95) | 2014 | Canada | RCT | Benzodiazepine therapy | Community pharmacy | 8 page booklet including a self-assessment about risks, evidence of potential harms, champion stories and a tapering protocol, and encouragement to discuss this decision with physician or pharmacist; Assessment of patient satisfaction about learning about the risks of benzodiazepine use |
| Taylor(96) | 2003 | United States of America | RCT | Antibiotics | Primary Care | Providing parents with educational materials about judicious antibiotics usage |
| Torke(97) | 2013 | United States of America | Qualitative interviews/focus groups | Cancer screening for persons with dementia | Outpatient screening | Assessed caregivers perspectives towards stopping screening for their relative and their knowledge of the potential risks |
| Torke(98) | 2013 | United States of America | Qualitative interviews/focus groups | Cancer screening | Outpatient screening | Explored patient knowledge and perspectives on cancer screening cessation |
| Tuso(99) | 2013 | United States of America | Editorial/commentary/letter-to-the-editor | Chronic dialysis | Hospital | Shared decision making surrounding the initiation of chronic dialysis and/or withdrawing treatment towards the end of life |
| van Leersum | 2019 | Netherlands | Narrative review | Low-value care in general | Not specified | Encouraging shared decision-making between patients and providers was one componet of a systems-level intervention to reduce unnecessary care |
| VBID Health(100) | 2017 | United States of America | Narrative review | Low-value care in general | Not specified | Patients pay higher co-payment for low-value services |
| Veet(101) | 2017 | United States of America | Cross-sectional | Daily labs in stable patients with no new symptoms | Hospital | Assess patient understanding and preference regarding daily labs |
| Volpp(102) | 2012 | United States of America | Editorial/commentary/letter-to-the-editor | Low-value care in general | Not specified | Patients pay higher co-payments for low-value services |
| Walsh-Childers(103) | 2018 | United States of America | Qualitative interviews/focus groups | Low-value care in general | Not specified | Assessing journalists understanding of over treatment and the reporting of over treatment in the media |
| Warner(104) | 2016 | United States of America | Cross-sectional | Computed tomography for headache (no red flags), antibiotics for upper respiratory tract infection | Primary Care | Assessed patient perceptions about low-value care using vignettes and potential responses from primary care providers |
| Weiner(105) | 2013 | United States of America | Website item/news | Low-value care in general | Not specified | Choosing Wisely campaign encourages patients to discuss whether or not they need this test |
| Wen(106) | 2015 | United States of America | Website item/news | Low-value care in general | Emergency Department | Shared-decision making to avoid unnecessary tests and procedures |
| Wennberg(107) | 2007 | United States of America | Narrative review | Low-value care in general | Hospital | Medicare beneficiaries use shared-decision making when facing the decision about discretionary surgery |
| Wheeler(108) | 2001 | United States of America | Cohort | Antibiotics | Primary Care | Educational videotape about the judicious use of antibiotics played in waiting rooms of paediatric physician offices |
| Wolfson | 2014 | United States of America | Narrative review | Low-value care in general | Not specified | Consumer Reports (a patient engagement partner) agreed to collaborate with the participating specialty societies to “translate” Choosing Wisely recommendations into consumer-friendly briefs (societies each distributed to 1 million consumers) |
| Wright(109) | 2017 | United States of America | Cohort | Intensive glycemic control | Primary Care | Shared decision making with patients to reduce the use of intensive glycemic control |
| ***Low-value care research*** | | | | | | |
| Allen | 2019 | Australia | Qualitative interviews/focus groups | Unnecessary CT scans | Primary care | Explored patient perceptions about shared decision-making about CT scan referral and use of the five Choosing Wisely questions with their GP |
| Althabe(110) | 2004 | Uruguay | Randomized controlled trial | Non-emergency caesarean section | Hospital | Patients receive a mandatory second opinion to reduce rates of unnecessary c-section; Outcomes included patient perceptions and satisfaction with the process of care and acceptability of the second opinion strategy |
| Ashurst(111) | 2014 | United States of America | Pilot study | Radiography | Emergency Department | Physicians used guidelines to decide if radiography was necessary and after discharge, patient satisfaction with care received was assessed on a 5 point Likert scale |
| Ashurst | 2014 | United States of America | Pilot study | Unnecessary radiography | Emergency Department | Patient satisfaction with care received through the de-adoption intervention was assessed on a 5-point Likert scale |
| Barker(112) | 2016 | India | Mixed methods | Antibiotics | Community pharmacy | Assessed community member knowledge of antibiotic misuse; asked community members how factors such as knowledge, access, and poverty related to antibiotic misuse |
| Blackmore(113) | 2011 | United States of America | Case report | Imaging tests for uncomplicated headache | Emergency Department | Assessing patient satisfaction after implementing an initiative to reduce imaging tests for uncomplicated headache |
| Canadian Psychiatric Association(114) | 2017 | Canada | Website item/news | Low-value tests and treatments in psychiatry | Not specified | A person with lived experience from the Canadian Mental Health Association was involved in the development of the Canadian Psychiatric Association’s Choosing Wisely List |
| Cho(115) | 2018 | United States of America | Consensus method | Low-value care in general | Hospital | Patient advocates where involved in co-creating a list of opportunities in overuse and underuse for improving value in hospital medicine |
| Chow(116) | 2015 | Canada | Consensus method | Low-value tests and treatments in Rheumatology | Not specified | Patient involvement in identifying and prioritizing 5 low-value practices for the Canadian Rheumatology Association's Choosing Wisely list |
| Daniel | 2018 | Canada | Editorial/commentary/letter-to-the-editor | Treatment of asymptomatic bacteriuria | Not specified | Engage patients and families on the quality improvement team to develop an initiative to reduce unnecessary treatment of asymptomatic bacteriuria |
| Engel-Rebitzer | 2019 | United States of America | RCT | Antibiotics for upper respiratory tract infections, diagnostic imaging for low risk ankle injuries | Emergency Department | Development of a two-minute video that used narrative or probabilistic elements to discuss decisions about low value care for the patients' presenting diagnosis;  Explored patient perspectives on methods for communicating with clinicians about reducing low-value care |
| Gesell | 2018 | United States of America | Qualitative interviews/focus groups | Unnecessary cardiac stress testing or angiography | Emergency Department | Patient advocate interviewed about the barriers and facilitators to an intervention to reduce unnecessary hospital admissions for cardiac stress testing or angiography |
| Goggin(117) | 2017 | United States of America | Randomized controlled trial | Antibiotics for acute respiratory tract infections | Outpatient clinic | Production of a video and brochure on antibiotic misuse; Conduct focus groups with parents to inform the design of the study and partner with a Parent Research Associate for the duration of the study; Parents will assess the experience of shared decision making, quality of parent-provider communication, and overall satisfaction with the visit |
| Gonzales(118) | 2005 | United States of American | Non-randomize controlled trial | Antibiotics for children with pharyngitis, antibiotics for adults with acute bronchitis | Primary care | Educational intervention: materials mailed to households, materials available in physicians offices, waiting rooms and exam rooms, describing the misuse of antibiotics; Focus groups with parents to develop and obtain feedback on paediatric educational materials prior to distribution |
| Hart | 2019 | Netherlands | Mixed methods | Overtreatment of glucose lowering treatments for Type 2 Diabetes | Primary Care | Patient questionnaire about discussions had and subsequent (or not) changes in medication since the intervention, and about their beliefs about the necessity of their medication;  Held focus groups with patients to explore their perspectives on the de-instensification intervention |
| Ho(119) | 2015 | United States of America | Consensus method | Low-value tests and treatments in newborn medicine | Not specified | Choosing Wisely list development: family representatives attending the Vermont Oxford Network Annual Congress were part of the national survey to identify low value practices in new born medicine for the consensus study as well as part of the panel for the consensus study. |
| Hofstede | 2019 | Netherlands | Qualitative interviews/focus groups | Vitamin D and B12 laboratory testing | Primary Care | Focus groups with patients to explore their perceptions of barriers and facilitators to reducing unnecessary vitamin testing in general practice |
| Iyengar | 2019 | United States of America | Cross-sectional | Low-value head CT scan for minor traumatic brain injury | Emergency Department | Cross-sectional survey assessed patient perspective on how financial incentives influence their willingness to forego low-value care |
| Kennedy | 2018 | United States America | Cross-sectional | Long term proton pump inhibitor use | Not specified | Patients provided input as to what would help them be willing to deprescribe from unnecessary PPIs |
| Lee(120) | 2017 | United States of America | Editorial/commentary/letter-to-the-editor | Low-value care in general | Not specified | Patient involvement in developing Choosing Wisely lists |
| Lemiengre(121) | 2014 | Belgium | Randomized controlled trial | Antibiotic prescribing for self-limiting disease | Primary care | Provide caregivers with written safety net advice on the antibiotic prescribing rate in acutely ill children not suspected of serious disease; Parents assessed clarity and readability of the leaflet used in the intervention; Evaluate parental perception of communication, parental satisfaction |
| Linder | 2018 | Australia | Editorial/commentary/letter-to-the-editor | Low-value care in general | Not specified | Patients can be involved in the evaluation of the Choosing Wisely Australia initiative through surveys to assess changes to awareness, attitudes and practice |
| Madill | 2019 | United States of America | Qualitative interviews/focus groups | Low-value care in general | Not specified | Patients evaluated early versions of an app to educate patients and provide patient-provider communication about low-back pain to reduce unnecessary tests and treatments |
| Maratt(122) | 2018 | United States of America | Systematic review | Low-value care in general | Not specified | Patient satisfaction, quality of life in relation to the outcome of an intervention to reduce low-value care |
| Maund | 2018 | United Kingdom | Systematic review | Antidepressants | Not specified | Explored patient perceptions of barriers and facilitators to deprescribing antidepressants |
| Melnick(123) | 2017 | United States of America | Feasibility study | CT use in Emergency Department | Emergency Department | Electronic tool to promote evidence-based conversation/shared decision making about the need for CT use; Patients were consulted during development of the tool |
| Mosconi | 2019 | Italy | Cross-sectional | Low-value care in general | Not specified | Explored the public's knowledge and perception of unnecessary tests and treatments |
| Muche-Borowski | 2018 | Germany | Consensus method | Low-value care in general | Not specified | Patient representatives were involved in the consensus process for prioritzing recommendations for overuse and underuse of health care services |
| Muscat | 2019 | Australia | RCT | Low-value care in general | Not specified | Patients evaluating if CW questions help them participate in shared-decision making to reduce low-value care |
| Nguyen(124) | 2017 | Canada | Consensus method | Low-value tests and treatments for Inflammatory Bowel Disease | Not specified | Choosing Wisely list development: 2 patient representatives participated in the face to face discussion day which was part of the third round of the modified delphi process, however the patients did not vote. This discussion moved from 10 candidate items to the final 5. |
| Omaki(125) | 2018 | United States of America | Pilot study | Opioids | Emergency Department | My Healthy Choice materials were produced to inform patients about environmental and personal risk factors associated with opioid use; Development of the program was informed by qualitative interviews with patients and refined and tailored by patients; Patient satisfaction with physician encounter and assessment of decision making process after sharing My Health Choices materials with physician |
| Omaki | 2019 | United States of America | RCT | Opioids for pain management | Emergency Department | Tailor patient education and decision aid to encourage patient to have an informed discussion about pain medication options with their ED provider;  Patient reported outcomes will include patient satisfaction, assessment of the decision-making process, decisional conflict and decisional regret |
| Perez | 2019 | United States of America | Mixed methods | Low-value care in general | Not specified | Assessed consumer awareness and decision making with value based insurance design |
| Perez | 2017 | United States of America | Community juries | Low-value care in general | Not specified | Session commenced with a review and discussion of an educational handout describing the problem of medical care overuse; the meaning of high- and low-value care; the reasons low-value care exists and its harms; and the medical research that forms the basis for evidence-based practice. This is followed by 3 scenarios of low value care and subsequent discussions;  Handout and scenarios were developed with input from a committee that was comprised of consumer advocates, health education experts, a patient representative and health policy leaders |
| Porath(126) | 2018 | United States of America | Cross-sectional | Diagnostic tests for low risk chest pain and mild traumatic brain injury | Emergency Department | Investigated individual level trade-offs between the benefits, risks, and cost of a low-value diagnostic test within hypothetical acute medical conditions commonly seen in an ED |
| Pramesh | 2019 | India | Consensus method | Low-value care in general | Not specified | Patient representatives were involved in the task force and consensus process for producing the Choosing Wisely list |
| Pruskowski | 2019 | United States of America | Systematic review | Inappropriate or unnecessary medications | Not specified | Included patient reported outcomes (eg, satisfaction) to evaluate deprescribing interventions |
| Rice(127) | 2018 | Canada | Qualitative interviews/focus groups | Blood transfusions for iron deficiency anemia (IDA) | Hospital | Patient focus groups to help develop patient education materials for reducing blood transfusions for IDA |
| Rietbergen | 2019 | Netherlands | Cross-sectional | Routine MRI and arthroscopy use in degenerative knee disease | Hospital | Survey asking patients what factors influence the implementation of CW recommendations for degenerative knee disease;  Patients were interviewed to inform the survey development (identify relevant factors to ask about in survey, for ex) |
| Shorten(128) | 2003 | Australia | Pilot study | Unnecessary caesarean section | Hospital | Decision aids for patients; Patients were involved in developing and refining the decision aid |
| Sharp | 2019 | United Kingdom | Consensus method | Low-value care in general | Outpatient clinic | Patient advisors were involved in the consensus process for a Choosing Wisely list |
| Stepanczuk(129) | 2017 | United States of America | Qualitative interviews/focus groups | Low-value care in general | Not specified | Patients described barriers and facilitators to accepting their physician’s recommendation to avoid a low-value practice |
| Trojanowski(130) | 2018 | United States of America | Qualitative interviews/focus groups | Antibiotics for urinary tract infections in long term care homes | Long term care | Focus groups with residents/families to understand barriers and facilitators to reducing antibiotic use in long term care homes; Focus groups with residents/families to assess the content, readability, and usability of an educational handout and video to address informational needs and improve communication between residents/families and care providers about why antibiotics will not be prescribed |
| van Egmond | 2018 | Netherlands | Qualitative interviews/focus groups | Follow-up care for low-risk basal cell carcinoma | Hospital | Explored patient perspectives about strategies for de-adoption of low-value follow-up care for low-risk basal cell carcinoma |
| ***Healthcare administration and policy*** | | | | | | |
| Bosslet(131) | 2015 | United States of America | Consensus method | Low-value care in general | Hospital | Engaging patients to develop societal policies and legislation about the appropriate boundaries of medical practice and futile care near the end of life |
| Costa | 2019 | Canada | Community Juries | Cancer drugs | Hospital | Explored the public's perspectives on the principles that should guide disinvestment decisions for cancer drugs in British Columbia |
| Degeling(132) | 2017 | Australia | Community Juries | Antibiotic overuse | Primary Care | Community juries to elicit views on the acceptability of proposed policy interventions designed to reduce the misuse of antibiotics in Australia |
| Degeling | 2019 | Australia | Community Juries | Low-value care in general | Not specified | Exploring the public's perspective on policymaking regarding screening overdiagnosis |
| Elshaug(133) | 2009 | Canada | Policy paper | Low-value care in general | Not specified | Involving patient and citizen groups to further a disinvestment agenda |
| Elshaug(134) | 2017 | Australia | Policy paper | Low-value care in general | Not specified | Public engagement to improve priority setting at the highest policy level, Patient engagement in clinical decision making to maximize value (especially when care is preference sensitive) |
| Hodgetts(135) | 2014 | Australia | Community Juries | Assisted reproductive technologies (ARTs) | Outpatient clinic | Forums with past patients and community members to discuss disinvestment and how to best distribute funding for ARTs |
| Ginsburg(136) | 2010 | United States of America | Editorial/commentary/letter-to-the-editor | Low-value care in general | Not specified | Assessed consumers' views on increased cost sharing or reduced coverage for less effective treatments |
| Healthcare Improvement Scotland(137) | 2013 | United Kingdom | Narrative review | Low-value care in general | Not specified | Public involvement in decision making relating to potential disinvestment in healthcare interventions and technologies |
| Hicks | 2018 | Canada | Narrative review | Low-value care general | Not specified | Patient representatives are involved in developing and executing the hospital's CW initiatives |
| Hollingworth(138) | 2015 | United Kingdom | Mixed methods | Low-value care in general | Not specified | Focus groups to explore patient perspectives on the candidate practice and potential disinvestment process |
| Lee(139) | 2017 | Canada | Quality Improvement | Benzodiazepines, stool softeners, proton pump inhibitors, multivitamins, antipsychotics | Hospital | Hospital's patient advisors were involved in implementing Choosing Wisely suggestions within the hospital |
| Lenaghan(140) | 1999 | United Kingdom | Narrative review | Low-value care in general | Not specified | Citizen's juries to engage the public in debates about the allocation of finite resources of health care |
| Minogue(141) | 2016 | United Kingdom | Narrative review | Low-value care in general | Not specified | Focus groups with public to discuss how they can be engaged in National Health Service financial decision making - patients felt they were the best judges of value and should be more involved in deciding which practices were of value |
| NHS England(142) | 2017 | United Kingdom | Website item/news | Low-value care in general | Primary Care | Public consultation on the development of national guidelines that identify medicines and treatments that should not be prescribed in primary care |
| NHS England(143) | 2017 | United Kingdom | Community Juries | Low-value drugs commonly prescribed in primary care | Primary Care | Surveys, webinars, engagement events to solicit feedback on a list of items that should not be routinely prescribed in primary care |
| Nicholls(144) | 2014 | Canada | Narrative review | Expanded newborn screening | Not specified | Public attitudes toward expanded newborn screening largely reflect professional and academic debates, with an emphasis on support for programs where the primary benefit is intervention for the child that will improve health outcomes; Research should to explore policy decision maker perspectives with respect to consent to newborn screening, and implications for parent understanding. |
| Ontario Citizen’s Council(145) | 2011 | Canada | Website item/news | Drugs on the Formulary for the Ontario Public Drug Programs | Not specified | Ontario Citizens' Council provided input and insight towards when drug products should be delisted from the formulary |
| Parchman(146) | 2017 | United States of America | Framework | Low-value care in general | Not specified | Patients were on the advisory committee that conducted a literature review, selected sites for environmental scan and created a framework for engaging providers and patients in reducing low value care |
| Rychetnik(147) | 2014 | Australia | Community Juries | Prostate cancer screening | Outpatient screening | Community jury on government investment in prostate cancer screening |
| Seo(148) | 2016 | South Korea | Systematic review | Low-value care in general | Not specified | Patient involvement in health technology reassessment by providing input and participating in the process |
| Street(149) | 2015 | Australia | Qualitative interviews/focus groups | Vitamin B12 pathology testing | Primary Care | Deliberative forum to develop criteria to support disinvestment of the publicly funded pathology test |
| Vernazza(150) | 2018 | United Kingdom | Consensus method | Low-value care in general | Dentistry | Involving patient representatives in the Program Budgeting and Marginal Analysis (PBMA) process to define areas for disinvestment |
| Watt(151) | 2012 | Australia | Community Juries | Assisted reproductive technologies, vitamin B12 and folate pathology tests | Not specified | Involving community members in the decision making process for disinvestment; asking community members about the barriers and facilitators of disinvestment |
